# Focus on materials science of topological insulators and superconductors

**DOI:** 10.1088/1468-6996/16/1/010301

**Published:** 2015-01-30

**Authors:** Akihiro Tanaka, Taizo Sasaki

**Affiliations:** National Institute for Materials Science, Japan

In a March 1997 article in *Physics Today* [1], Gerald Mahan, Brian Sales and Jeff Sharp write on what they deem a ‘new approach to an old problem’. The subject is thermoelectric materials, which is indeed an old problem, predating the inception of modern solid state physics itself: thermoelectric refrigeration using wires made of bismuth and antimony, the article points out, dates back to 1838. A major pivotal point came in the 1950s with the recognition that semiconductor-based home refrigerators may well become a reality, and the flurry of R&D activities that ensued turned up among other findings a prominent material, the Bi_2_Te_3_/Sb_2_Te_3_ alloy which operates well at room temperatures. Mahan *et al* make the case that the time is ripe to take another stab at such materials, what with the growing concerns on environmental issues, not to mention the considerable advance in synthesis techniques and theoretical methods now at the researcher’s disposal. They assert that by enlisting the full force of these latest know-hows, the ‘old problem’ can be turned into a promising playground for new applications.

As much as these views are farsighted, it turns out that history had yet another twist in store for these and closely related materials, coming from a totally different field and in a manner completely unanticipated at the turn of the century. Semiconductors with strong spin–orbit interactions came into focus among condensed matter physicists seeking new guiding principles in their quest for materials with spintronic applications. This line of effort culminated in a number of important theoretical papers published around the year 2005 on the spin quantum Hall state, a new state of matter predicted to transport spin (but not charge), which can form in electron systems residing in a two-dimensional environment such as graphene. This was quickly followed by an experimental verification of the state in quantum dots, as well as the proposal of a 3d counterpart which is now commonly called a topological insulator—actually, there are several other classes of topological insulators, but here we shall adhere to popular usage. As far as material properties go, these insulators are basically featureless in the bulk, yet they have a highly unusual surface metallic state, featuring a Dirac fermion-like mode (or an odd number of them) and an assortment of novel quantum effects which cannot occur in the bulk of a detached 2d system. Interestingly, much of the experimental premises in this development cover common grounds with the materials related to in the opening paragraph—and for good reasons. While our focus in this issue is not on thermoelectric properties, it is nevertheless instructive to ponder a bit on this point. First of all, from a materials design point of view, both purposes tend to favor a modest energy gap and heavy elements. However it is perhaps even more essential here to note the distinguishing feature of topological insulators: they are *symmetry-protected* and *topology-protected*. They are symmetry-protected because unless time reversal symmetry is broken (e.g. by turning on a magnetic field), this quantum state cannot be destroyed by perturbations. They are topology-protected since this symmetry-protection owes to the presence of a topological winding number linked to this quantum state, which cannot change unless something drastic enough to diminish the energy gap altogether is done to the system. This, in turn implies that the surface state unique to topological insulators and the transport properties that they sustain are also largely immune to disorder. Needless to say, the same cannot be said of surface metallic states which occur in conventional materials—there, a minute disorder will have an immediate effect. It thus follows that the transport channels—including those for heat transport—are unusually resilient to impurities and other agents which would otherwise degrade their characteristics. Furthermore, crystalline defects, which may typically be viewed as a sort of sample surface, can also give rise to additional conducting channels, which is another highly unconventional aspect of this state. This has prompted theorists to propose e.g. that a population of dislocations embedded in a topological insulator will enhance the figure of merit of the material [2].

It is intriguing that the building blocks of the topological insulator story simply boil down to elements of basic quantum mechanics (Kramers theorem and Berry phases) and traditional electron band theory, and yet they conspire to predict the existence of a band insulator state belonging to a phase which must be regarded as being distinct from the textbook insulator. The development of the past decade (which is still evolving) therefore constitutes a prime example of a ‘new approach to an old problem’, with the new aspect being that some topological concepts have been incorporated into electron band theory. It is not difficult to envision solid state physics textbooks being updated in the near future to take these fundamental discoveries into account.

There are by now a number of authoritative review articles [3] and journals with special issues devoted to topological insulators [4], to which we refer readers for good introductions to the subject. Here we have tried to organize a unique collection of articles written by prominent players in this emerging field under the conviction that (1) it may well be that we are still at the tip of the iceberg, and this issue should accordingly be compiled with a wide scope, allowing the reader to see the larger landscape of the subject, and (2) as the research front is expanding with the concerted efforts of condensed matter physicists and materials scientists, we should take an interdisciplinary stance in selecting the topics to be covered. Readers will find that the subjects taken up indeed have a wide spectrum. Topics include: the synthesis of topological insulators, theory/experiments on novel transport in Weyl semimetals (the 3d analog of graphene), the status of the Si counterpart of graphene, a feasible scheme for generating/manipulating the much sought for Majorana fermions in topological superconductors (the superconducting cousin of topological insulators), proposals on transforming conventional materials into topological insulators using laser lights, a report on topological insulators in action in phase change memories and their potential applications, the finite-size effects unique to topological insulators which can be turned into an advantage in nanostructures, and the optical analog of topological states of matter using photonic crystal waveguides. It is our intention to convey to the reader the breadth of the phenomena which come under the umbrella of topological states of matter.

We hope that we have been successful, and wish to thank the authors for their valuable efforts.
